# Effects of reducing excess dental adhesive on bacterial adhesion in the bracket periphery

**DOI:** 10.1007/s00784-023-04924-4

**Published:** 2023-02-21

**Authors:** Christoph-Ludwig Hennig, Bijan Blochberger, Judit Symmank, Ánn Nitzsche, Sandor Nietzsche, Frank Steiniger, Marco Dederichs, André Güllmar, Markus Reise, Ulrike Schulze-Späte, Bernd Sigusch, Collin Jacobs

**Affiliations:** 1 Department of Orthodontics, Center of Dental Medicine, University Hospital Jena, An Der Alten Post 4, 07743 Jena, Germany; 2 Electron Microscopy Center, University Hospital Jena, Ziegelmühlenweg 1, 07743 Jena, Germany; 3 Department of Prosthodontics, Center of Dental Medicine, University Hospital Jena, An Der Alten Post 4, 07743 Jena, Germany; 4 Department of Conservative Dentistry and Periodontology, Center of Dental Medicine, University Hospital Jena, An Der Alten Post 4, 07743 Jena, Germany; 5 Section of Geriodontics, Department of Conservative Dentistry and Periodontology, Center of Dental Medicine, University Hospital Jena, An Der Alten Post 4, 07743 Jena, Germany

**Keywords:** White spot lesion, Flash-free brackets, Biofilm formation, Bracket periphery, Bacterial adhesion

## Abstract

**Objectives:**

White spot lesions are one of the most common side effects of orthodontic therapy with a multibracket appliance and may indicate a preliminary stage of caries, also known as initial caries. Several approaches may be utilized to prevent these lesions, such as reducing bacterial adhesion in the area surrounding the bracket. This bacterial colonization can be adversely affected by a number of local characteristics. In this context, the effects of excess dental adhesive in the bracket periphery were investigated by comparing a conventional bracket system with the APC flash-free bracket system.

**Materials and methods:**

Both bracket systems were applied to 24 extracted human premolars, and bacterial adhesion with *Streptoccocus sobrinus* (*S. sobrinus)* was performed for 24 h, 48 h, 7 d, and 14 d. After incubation, bacterial colonization was examined in specific areas by electron microscopy.

**Results:**

Overall, significantly fewer bacterial colonies were found in the adhesive area around the APC flash-free brackets (*n* = 507 ± 13 bacteria) than the conventionally bonded bracket systems (*n* = 850 ± 56 bacteria). This is a significant difference (**p = 0.004). However, APC flash-free brackets tend to create marginal gaps with more bacterial adhesion in this area than conventional bracket systems (*n* = 265 ± 31 bacteria). This bacterial accumulation in the marginal-gap area is also significant (*p = 0.029).

**Conclusion:**

A smooth adhesive surface with minimal adhesive excess is beneficial for reducing bacterial adhesion but also poses a risk of marginal gap formation with subsequent bacterial colonization, which can potentially trigger carious lesions.

**Clinical relevance:**

To reduce bacterial adhesion, the APC flash-free bracket adhesive system with low adhesive excess might be beneficial.

APC flash-free brackets reduce the bacterial colonization in the bracket environment.

A lower number of bacteria can minimize white spot lesions in the bracket environment.

APC flash-free brackets tend to form marginal gaps between the bracket adhesive and the tooth.

## Introduction

White spot lesions and caries in the bracket environment are common side effects of orthodontic treatment with a multibracket appliance [1]. White spot lesions may indicate a preliminary stage of caries, also known as initial caries. These lesions are caused by tooth surface demineralization, which is triggered by acids in the oral biofilm that is formed when caries-associated bacteria metabolize carbohydrates. These pH-value changes in the biofilm and the modifications in the oral environmental conditions lead to cariophatogenic microflora, which is associated with increased colonization of acidogenic and aciduric bacteria. These again increase the risk of caries and tooth decay [2]. In particular, the facultative anaerobe *Streptococcus sobrinus* (*S. sobrinus*) is known for early colonization during the formation of white spot lesions. This bacterium, a part of the oral flora, accumulates within a few hours after tooth cleaning [2]. S. *sobrinus* is therefore one of the first bacteria to colonize surfaces and provides the basis further bacteria adhesion. As one of the early colonizers of dental surfaces, dental materials, and orthodontic appliances in the oral cavity, S. *sobrinus* is suitable for use in examining surfaces for bacterial accumulation in the oral cavity [3, 4]. Furthermore, *S. sobrinus* is known to produce acids and polysaccharides that adhere particularly well to tooth surfaces in the context of biofilm formation. Consequently, they form the basis for the multiplication of bacteria. In addition to the bacterial occurrence, the time, biofilm formation, and accumulation of the biofilm in the bracket environment are crucial determinats of the formation and severity of white spot lesions [5–8].

In general, the incidence of the formation of new carious lesions in orthodontic patients has been reported as exceeding 45%, and the overall prevalence of caries in patients undergoing orthodontic treatment has been reported at more than 68% [9]. Several options to reduce the risk for white spot lesions and caries during orthodontic treatment exist. First, orthodontists should encourage their dental patients to practice comprehensive and careful oral hygiene. For improvement, professional dental cleanings should be offered, fluoride-containing products should be used, or sealants for the enamel surface could be applied [1, 10]. Reducing biofilm formation in the bracket environment is the best method for reducing the occurrence and severity of white spot lesions and caries. In this context, the inclusion of anti-bacterial compounds in the bracket adhesive or the complete removal of excess adhesive around the bracket seems to be beneficial [11–13]. Thus, a smooth conjunction between brackets and enamel might be useful for the abatement of bacterial adhesion in this area. However, bacterial formation in and around margin gaps may be another problem. A margin gap is defined as a gap area between the adhesive area on the underground of the bracket and the tooth surface. A new adhesive technology (APC Flash-Free Adhesive Coated Appliance System, 3 M Unitek, Monrovia, USA) has eliminated the need for the removal of excess adhesive material during bracket bonding compared to the standard bonding technique. The adhesive layer is incorporated at a fiber matrix on the bracket base that is precoated by the manufacturer. Various aspects such as bonding time, adhesive remnants after debonding, the morphology of resin excess, and the bracket–adhesive–tooth interface are advantages of APC flash-free brackets over conventional brackets used with regular adhesive [15–19]. The aim of the present study was to investigate the bacterial adhesion and colonization in vitro in the adhesive region of APC flash-free brackets compared to conventionally bonded brackets. We hypothesized that a lower excess of adhesive protects the tooth surface against bacterial adhesion, and therefore fewer bacteria will accumulate in the bracket periphery. The reduced bacterial colonization should thus also minimize the side effects of white spot lesions. This could be an advantage of APC flash-free brackets.

## Materials and methods

### Tooth selection and preparation

Extracted teeth were collected from dental and oral surgery practices and subsequently sorted for suitability for the study. The teeth were manually cleaned with water after extraction, disinfected in alcohol for 2 min, and stored in 0.9% sodium chloride solution. Since an intact enamel is essential for the experimental procedure, all teeth with caries, fluorosis, enamel cracks, white spot lesions, anatomical anomalies, enamel spalling due to extraction, and iatrogenic restorations were excluded. Only premolars that were examined for an intact and caries-free enamel surface and freedom from enamel structure defects were selected. Thus, we included teeth with healthy and natural enamel in the study. The patients gave their consent to release the extracted teeth for the study. The consent of the ethics committee of the medical faculty at the University of Jena was obtained with ethic number 2021–2486_1.

In total, 24 extracted caries-free human premolars were equally divided into two groups—Group A: APC flash-free brackets and Group B: conventional brackets. All brackets used were clarity ceramic brackets (3 M Unitek, Monrovia, USA).

### Bracket-bonding protocol

For both groups, we used the following bracket-bonding protocol. All brackets were bonded by an orthodontist. Under a sterile workbench, the teeth were cleaned with a fluoride-free polishing paste (Proxyt, Ivoclar–Vivadent, Schaan, Liechtenstein) and a brush. After drying, all teeth were uniformly prepared with Transbond Plus Self Etching Primer (3 M Unitek, Monrovia, USA). The mixture was prepared exactly according to the manufacturer’s instructions, and Transbond Plus was applied to the area of the tooth intended for the bracket and massaged in for 5 s. Next, the bonding was lightly blown for approximately 2 s and then light polymerized for 20 s. An Acteon Mini LED polymerization light was used for this purpose. After that, one bracket (Group A: APC flash-free brackets; Group B: conventional brackets) per tooth was glued on the buccal side and light polymerized for 20 s. The brackets were placed directly along the longitudinal axis of the tooth on the vestibular side, 4 mm cervical from the cusp tip. In the case of Group B, Transbond adhesive (3 M Unitek, Monrovia, USA) was used; the Group-A brackets are self-adhesive. In the case of the conventionally glued brackets, the excess adhesive was generally removed with a probe, as typically performed, to smooth out the adhesive area and reduce bacterial adhesion.

### Bacterial culture

**Fig. 1:**
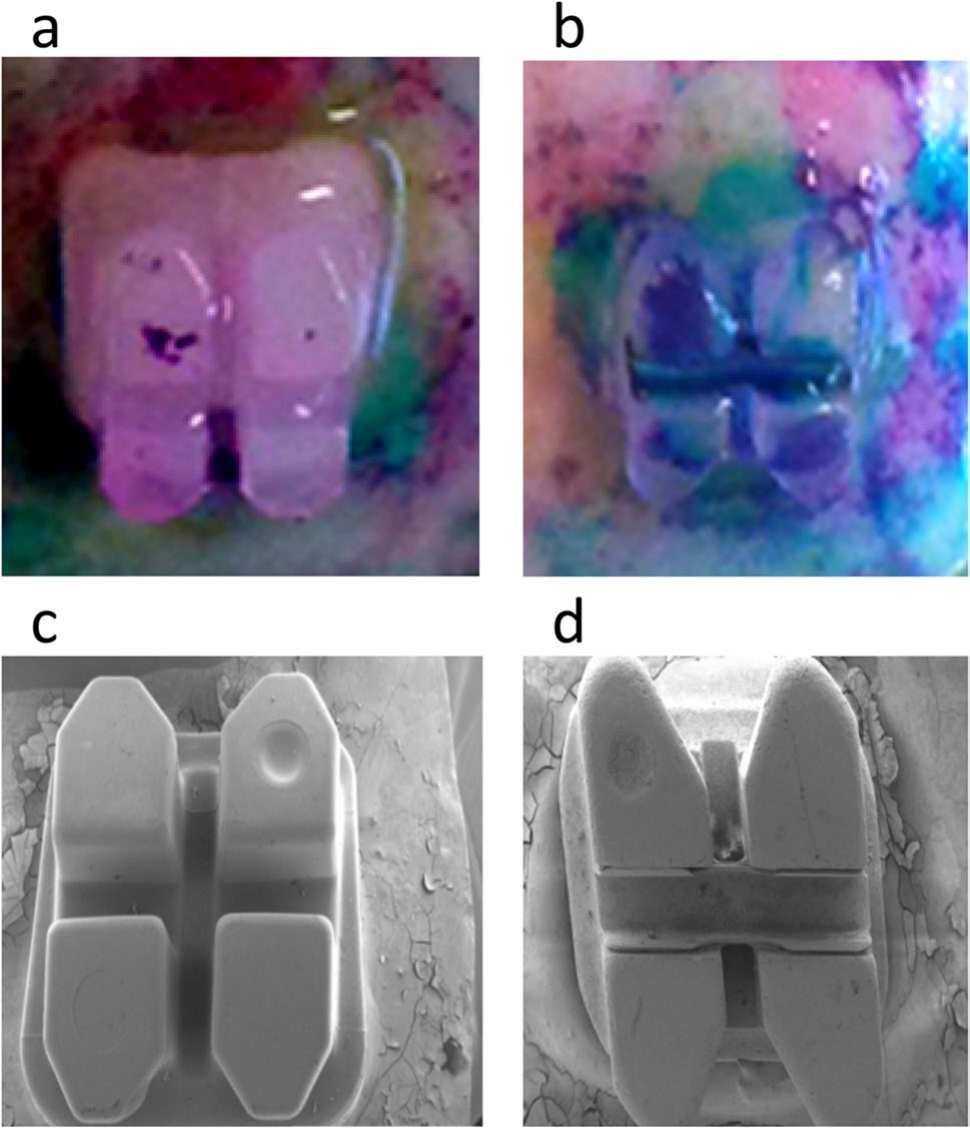
Staining of brackets and teeth with **a**, **b** Mira-2-Ton solution and REM recording of the **c** APC flash-free brackets and **d** conventionally bonded standard brackets after 48 h in bacterial culture. The photograph shows a higher occurrence of bacteria in **b** conventionally bonded standard brackets than in **a** APC flash-free brackets. The REM of the **d** standard brackets supports this achievement because of the bacterial adhesion, especially at the right side of the picture

At first, *Streptococcus sobrinus* (*S. sobrinus*) was cultured in Schaedler Bouillon (Carl Roth GmbH, Karlsruhe, Germany) and used in the experimental setup after reaching an optical density of 0.77. After that, the teeth were incubated with bacterial suspension in 6-well plates containing 1 ml bacterial suspension on 10 ml Schaedler Bouillon (Carl Roth GmbH, Karlsruhe, Deutschland) and incubated in an anaerobic box (MKIII Fa., Meintrup DWS) at 37 °C, 80% N_2_, 10% CO_2_, and 10% H_2_, and then removed at different intervals (24 h, 48 h, 7 d, and 14 d, respectively). For the pre-, qualitative, and quantitative analysis of the bacterial colonization and to determine where and how many bacteria are found in a direct comparison between the bracket systems, the test specimens were additionally stained with Mira-2-Ton solution (Miradent, Duisburg, Germany) (Fig. 1).

### Electron microscopy

For electron microscopy, tissue fixation was performed for 20 min with 4% glutaraldehyde. After that, samples were washed three times in 0.1 M cacodylate buffer, and dehydration was performed with an ethanol series of 30%, 50%, and 70% for 10 min each and 70% ethanol overnight at 4 °C. Subsequently, the samples were scanned with a Zeiss (LEO) 1450 VP (Variable Pressure) raster electron microscope (REM) with X-ray microanalysis (Bruker Quantax EDS 100; resolution: 4 nm; image documentation: digital microscopy).

### Image analysis and statistical evaluation

**Fig. 2:**
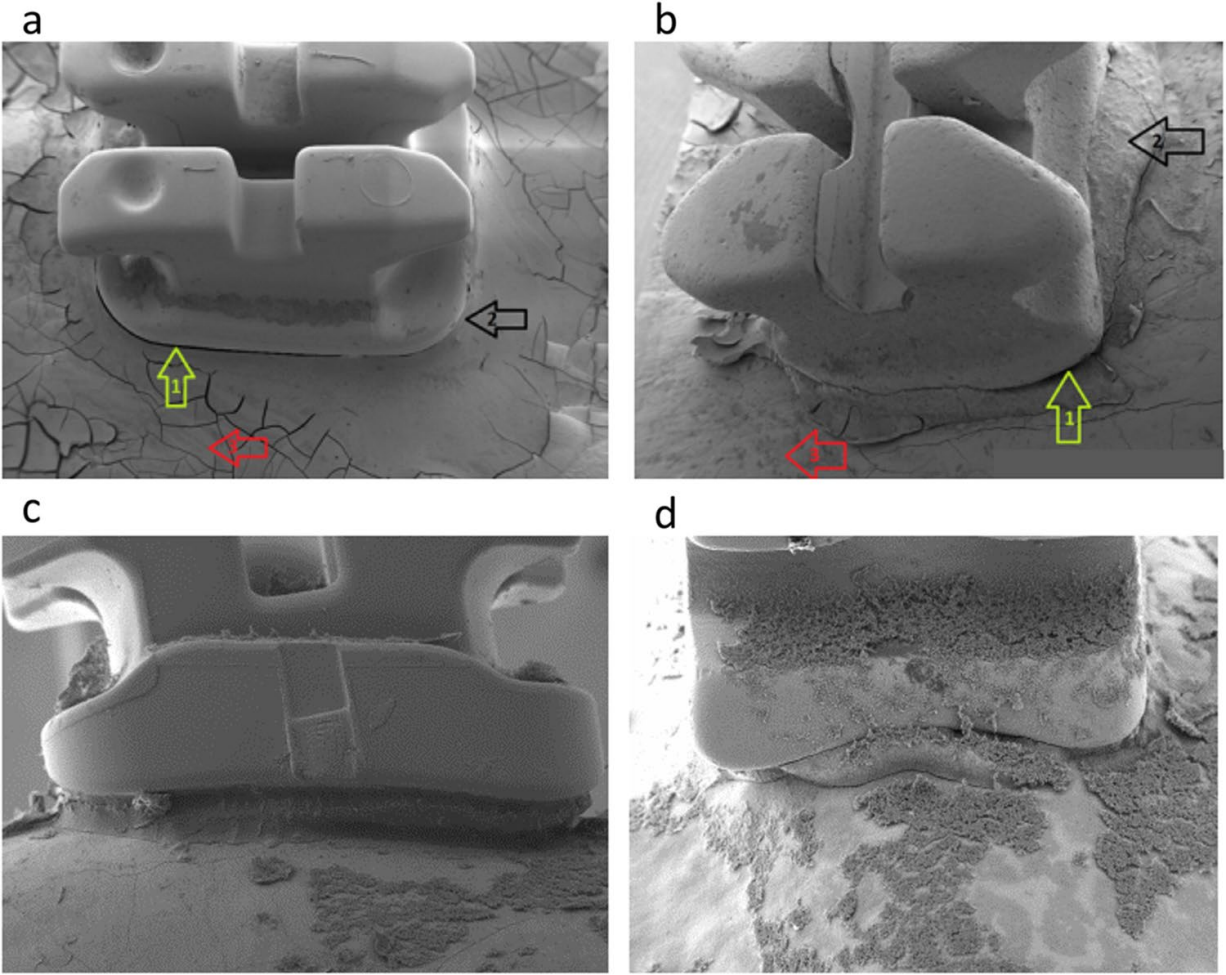
Scanning electron microscope images in overview (with × 20 magnification) of the **a** APC flash-free bracket and the **b** standard bracket. 1 = adhesive area, 2 = bonding area, 3 = margin gap. Side view (with × 30 magnification) of the adhesive range and the margin gap of Group A (**c** APC flash-free bracket) and Group B (**d** standard bracket) after 48 h in *S. sobrinus* bacterial culture

**Fig. 3:**
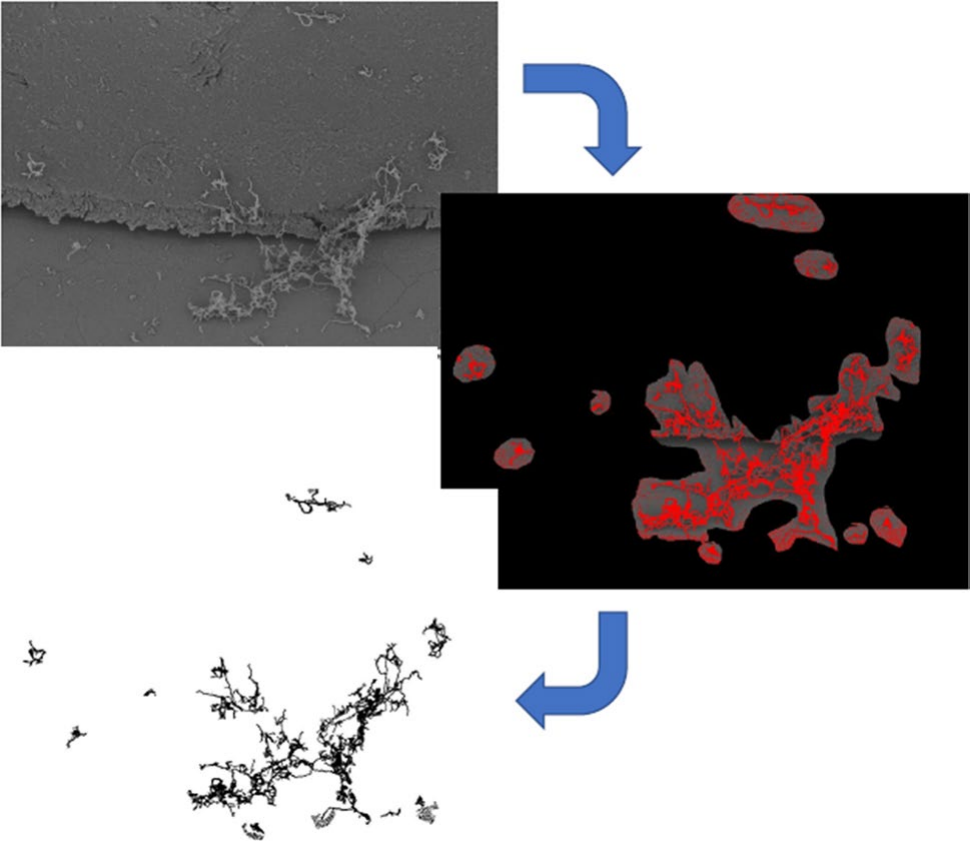
Bacterial analysis and counting in the bracket environment with the program ImageJ. The REM image was evaluated with the “Threshold” function, and the particles were counted with the feature “Analyze Particles”

The analysis of the electron microscopic images was performed with ImageJ (public domain, Open Source). For further investigations, the clinically important sectors of “adhesive area,” “margin gap,” and “bonding area” were defined, and sectors of 26.6 μm × 20 μm were examined (Fig. 2). The adhesive area is the region of adhesive between the bracket and the bonding coated tooth and also the excess flash around the bracket. The margin gap is the area between the adhesive and the tooth. The bonding area is the direct range of the tooth around the bracket, where excess bonding from the attachment of the multibracket appliance remains. For each bracket, three sectors in the adhesive area, margin gap, and bonding area were defined on each side. The data were collected using ImageJ (public domain, Open Source). To count the bacterial colonies, the “Threshold” function with equal settings was applied to the microscopic images, and the resulting particles were analyzed with the “Analyze Particles” function (size: 0–infinity, circularity: 0.00–1.00 show: masks, display results, clear results, summarize) (Fig. 3). In addition, a control area with maximum bacterial growth was counted both via ImageJ and manually.

Statistical evaluation was performed using GraphPad Prism (GraphPad Software Inc., San Diego, CA, USA). Statistical data significances were measured using a nonpaired *t*-test, with *p*-values of less than 0.05 considered statistically significant.

## Results

After review and analysis of the test specimens, only those incubated for 48 h in the anaerobic box were examined further. At all earlier and later sampled time points, too few bacteria were detectable due to possible substrate saturation.

**Fig. 4:**
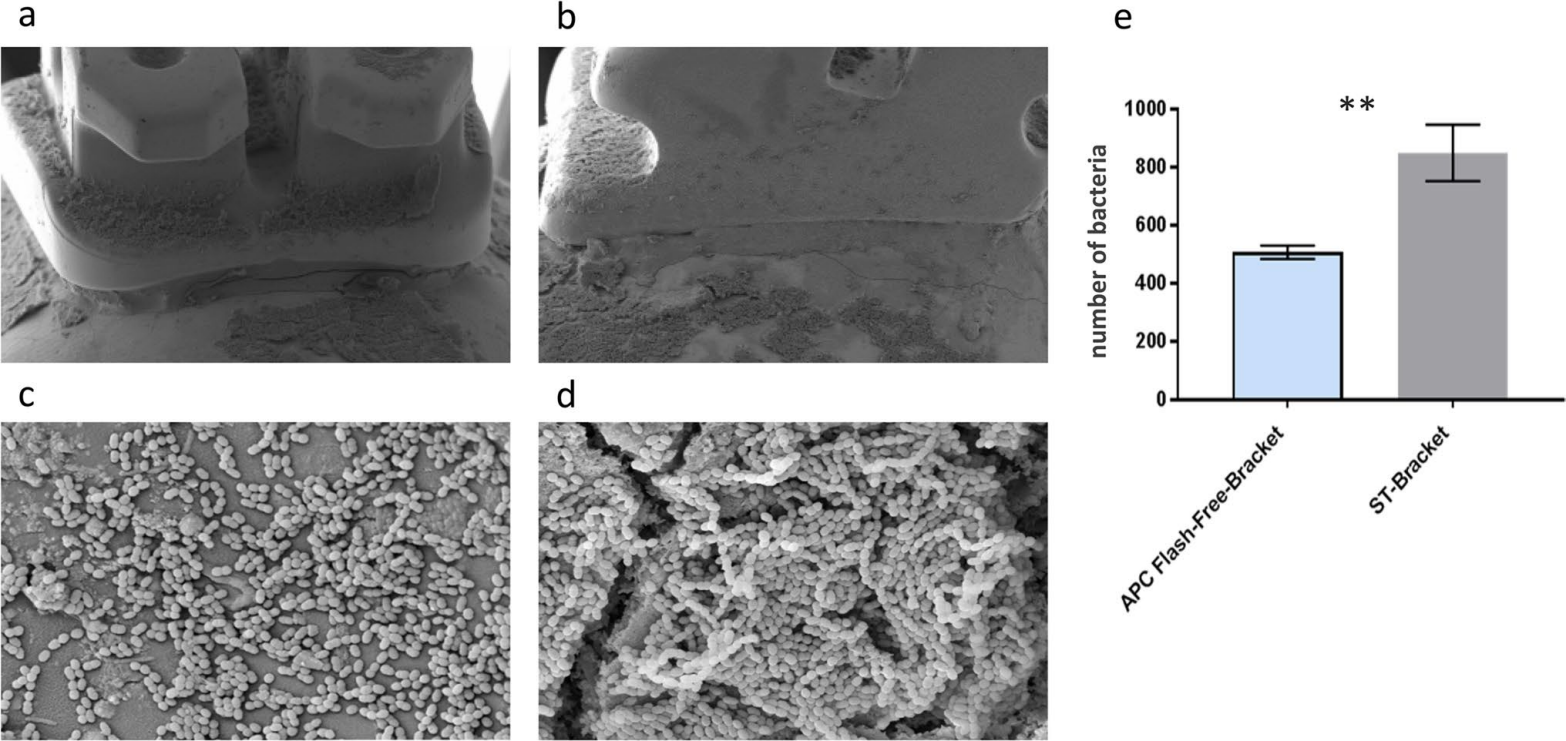
Electron microscope side view image (in × 30 magnification) of the “adhesive area” to be found in the **a** APC flash-free bracket and the **b** conventionally bonded standard bracket. Bacteria accumulation of *S. sobrinus* in the adhesive area of the **c** APC flash-free bracket and the **d** conventionally bonded standard bracket (in × 400 magnification). **e** Statistical results of the average bacteria number in the “adhesive area” in a 26.6 μm × 20 μm range show a significantly higher bacterial occurrence (**p = 0.004) in the conventionally bonded standard brackets (ST-Bracket) (*n* = 850 ± 56 bacteria) than in the APC flash-free brackets (*n* = 507 ± 13 bacteria)

The mean with standard deviation of bacteria in the adhesive area in Group A (APC flash-free brackets) was *n* = 507 (± 13) bacteria and in Group B (conventionally bonded standard brackets) was n = 850 (± 56) bacteria (Fig. 4), which is a significant difference (**p = 0.004). From this follows that the Group-A brackets showed lower bacterial adhesion within the first 48 h compared to the Group-B brackets (Fig. 4).

**Fig. 5:**
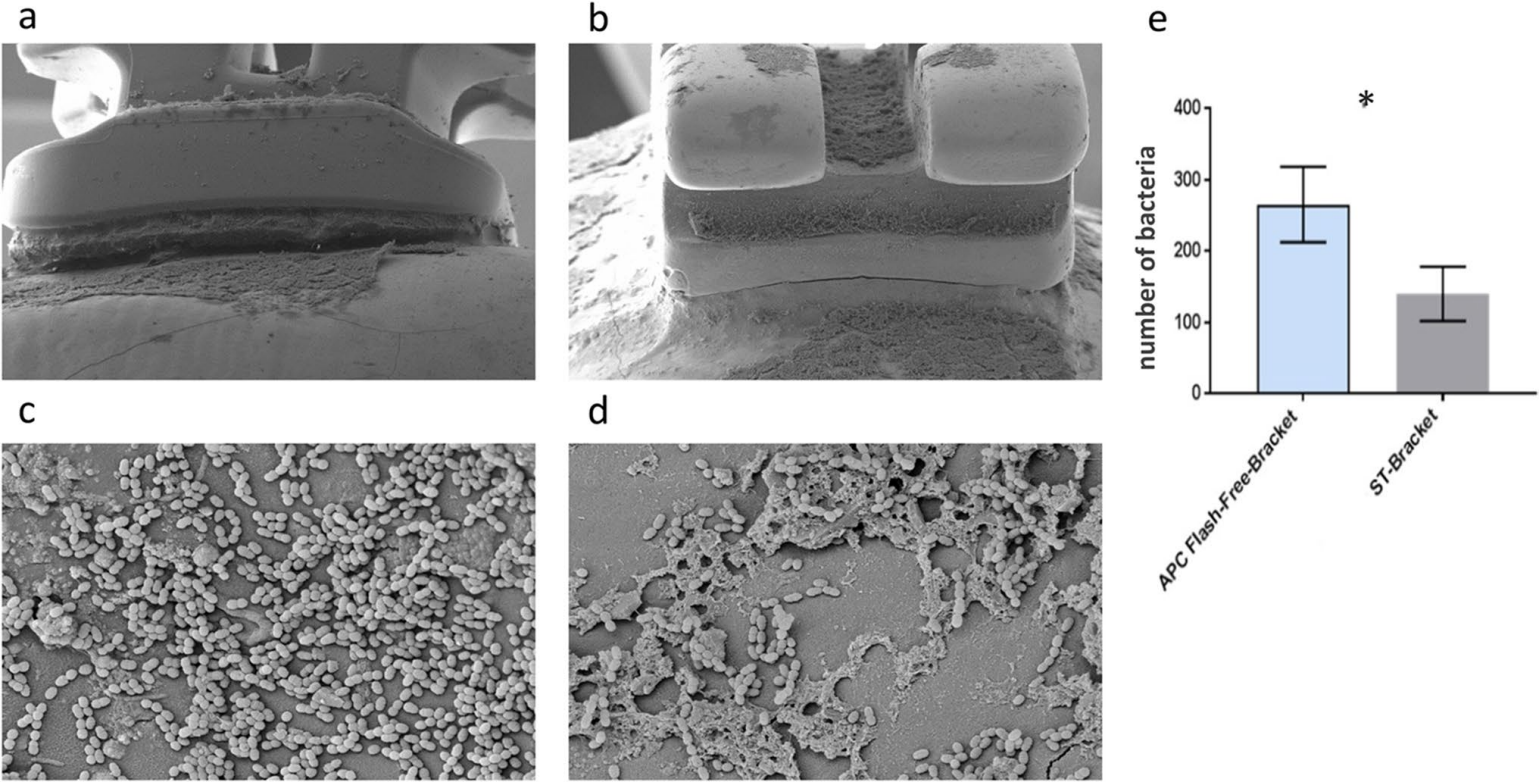
Electron microscope side view image (in × 30 magnification) of the margin gap to be found in the **a** APC flash-free bracket and the **b** conventionally bonded standard bracket. Bacteria accumulation of *S. sobrinus* in the margin gap of the **c** APC flash-free bracket and the **d** conventionally bonded standard bracket (in × 400 magnification). **e** Average number of bacteria in the “margin gap” in a 26.6 μm × 20 μm area shows a significantly higher occurrence (*p = 0.029) in the APC flash-free brackets (*n* = 265 ± 31 bacteria) than in the conventional standard brackets (ST-Bracket) (*n* = 140 ± 22 bacteria)

Furthermore, a significant difference (*p = 0.029) was also observed in the margin gaps. The average number of bacteria in the group of APC flash-free brackets (*n* = 265 ± 31 bacteria) was higher than in the group of standard brackets with conventional adhesive (*n* = 140 ± 22 bacteria), which was evident in the REM image (Fig. 5).

**Fig. 6:**
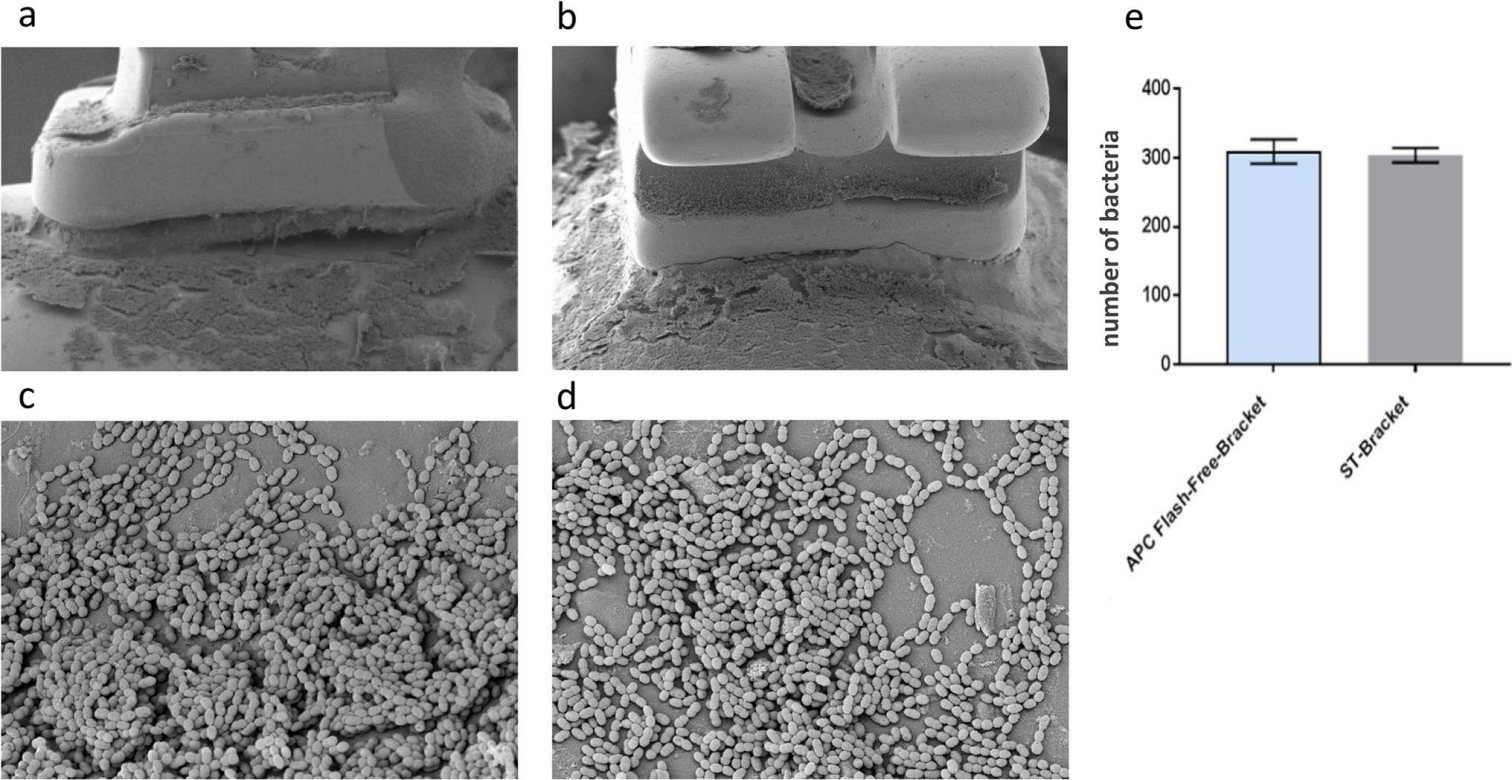
Electron microscope side view image (in × 30 magnification) of the bonding area to be found in the **a** APC flash-free bracket and the **b** conventionally bonded standard bracket. Bacteria accumulation of *S. sobrinus* in the bonding area of the **c** APC flash-free bracket and the **d** conventionally bonded standard bracket (in × 400 magnification) **e** Average number of bacteria in the”bonding area” in a 26.6 μm × 20 μm area does not show a significantly higher occurrence in the APC flash-free brackets (*n* = 309 ± 10 bacteria) than in the conventionally bonded standard brackets (St-Bracket) (*n* = 304 ± 6 bacteria)

In the bonding area, the average number of bacteria was not significant. The amount in the APC flash-free brackets (*n* = 309 ± 10 bacteria) and in the conventionally bonded standard brackets (*n* = 304 ± 6 bacteria) was comparably high (Fig. 6).

In summary, our study showed significantly reduced *S. sobrinus* colonization in the adhesive surface in the case of the APC-free-flash brackets compared to conventional brackets, although the number of bacteria in the marginal gap increased.

## Discussion

The prevention of white spot lesions and the enamel demineralization of patients with orthodontic appliances is essential [1, 20, 21]. This prevention of caries formation during orthodontic therapy can be a result of the bacterial milieu, and the number of bacteria in the bracket environment [10, 22]. When fewer bacteria colonize, fewer carbohydrates are metabolized, resulting in fewer acids that could potentially attack the primarily healthy enamel [23, 24]. The structures and surfaces in the mouth are predestinated for the colonization of bacteria. Surface roughness and size play decisive roles in plaque accumulation. Various methods have been established to protect enamel during multibracket treatment. In 2015, Montasser et al. investigated the protection of enamel by sealants during multibracket treatment [10]. However, the adhesive and texture in terms of structure and roughness are understood to have special roles in bacterial accumulation. In 1975, Weitmann et al. investigated bacterial plaque accumulation in composite restorations, illustrating the differences of various composite/adhesive excesses and their finishing by polishing in relation to bacterial adhesion [22]. The studies by Sukontapatipark et al. (2001) and Försch et al. (2016) showed that this could also play a role in the development of white spots in orthodontics. Sukontapatipark et al. (2001) dealt with the microscopic examination of excess adhesive in standard brackets. In these experiments, the brackets were bonded to premolars in vivo and left in the oral cavity for 1, 2, or 3 weeks. After this, the brackets were extracted and examined using electron microscopy. Sukontapatipark found that the characteristics of dental composite excess in the bracket area were a decisive factor for plaque accumulation [25]. Försch et al. (2016) compared the difference in excess adhesive between standard brackets and APC flash-free brackets. Försch et al. investigated various aspects of APC flash-free brackets such as time for bonding, the stereomicroscope evaluation of excess adhesive, color penetration, and the adhesive remnant index score after debonding. This was the first study to microscopically evaluate the composite excess, showing that it was significantly lower in APC flash-free brackets at 81.66 to 166.27 µm compared to standard brackets with 690.81 to 988.53 µm [15]. Thus, excess adhesive in orthodontic attachments should be kept at a low level, and each bracket should still be secured to the tooth as tightly as possible [24]. Moreover, the characteristics of APC flash-free brackets were investigated in 2015 and 2018 by Grünheid et al. in terms of debonding and adhesive clean-up as well as bracket failure rates [26, 27]. In 2015, Grünheid found that there was no significant difference in debonding between standard brackets and APC flash-free brackets, but the amount of adhesive remnant was significantly higher for the APC flash-free brackets, while there was no significant difference in adhesive cleaning time [26]. Furthermore, Grünheid (2018) investigated the bonding time and bracket failure rates in vivo between standard brackets and APC flash-free brackets. It was significantly shown that the bonding time per tooth was 37.7% shorter for the APC flash-free brackets, but the bracket failure rates in 1 year were for this part 2.8% higher than in standard brackets [27]. Similarly, the study by Soliman et al. (2022) showed the bracket failure rate of APC flash-free brackets compared to other bracket and adhesive systems [28]. Overall, the studies found that the low level of excess adhesive in APC flash-free brackets shortens the debonding time and clean-up time, but the bracket failure rate is higher.

In summary, our study showed the difference in bacterial accumulation to be a function of adhesive excess, using both standard brackets and APC flash-free brackets in the context of electron microscopy. Self-adhesive brackets (Group A: APC flash-free brackets) are attached without any extra adhesive, so they have significantly less glue overflow around the adhesive area and therefore offer less habitat for bacteria. As also proven by the results, the bacteria adhesion in the adhesive area was significantly lower than with standard brackets. However, conventionally bonded standard brackets are attached with extra adhesive on the tooth surface, and the excess must be removed with a probe by the orthodontist. Therefore, the edges in the adhesive area were much smoother in Group B (conventionally bonded standard brackets) than Group A (APC flash-free brackets). This is why the standard brackets created shallower margin gaps than the APC flash-free brackets. The results also showed that significantly fewer bacteria settled in the margin gap area in the case of the standard brackets than in the case of the APC-flash-free brackets. Because of the identical bonding systems and technique, the bonding area showed no significant difference between the two groups.

A limiting factor of the study is that it shows the bacterial count in the electron microscope purely optically. Thus, only the locations of bacterial colonization in the bracket area were defined. The activity of bacterial colonization was not considered. Therefore, it is possible that, although there was a high level of bacterial colonization at some locations in the bracket environment, this had no pathogenic relevance because the bacteria were inactive or had already died. Regarding this, further microbiological methods are recommended for future studies. Moreover, a disadvantage of the study is that in course of the various test times, only the period after 48 h of incubation was examined in detail. Since according to random checks and analysis, most bacterial adhesion occurred at this time. The substrate appears to be saturated after a prolonged incubation, so the bacteria could not proliferate longer. The reason for this could be that in the initial period, the bacterial count must first increase, and a certain milieu must first be present for bacterial proliferation to occur. At later times, the saturation with the nutrient substrate seems to have been insufficient, so that no further reproduction of the bacteria took place. König et al. (1971) would confirm this hypothesis since bacterial numbers decrease when substrate decreases [29]. Another cause could be that the bacteria displace or overlap each other on the surface and more bacteria cannot be detected with the present optical evaluation method. A better representation of bacterial accumulation would be made by examining multiple time points and investigating the activity of the bacteria present at those time points.

Our study aimed to show how the self-adhesive property of APC flash-free brackets affects the bacterial accumulation in the bracket environment compared to conventionally bonded standard brackets. It was shown that due to the reduction of the adhesive area of the APC flash-free brackets, fewer bacteria accumulated in the adhesive area, and thus fewer caries lesions are likely to be created around the bracket. One the other hand, a higher occurrence of margin gaps in the APC-flash-free brackets was observed. There were more marginal cleavages, giving bacterial colonies the opportunity for plaque formation. This may be due to insufficient adhesives in the pre-assembled fleece of the APC flash-free brackets or because the same fleece was used for all APC flash-free brackets regardless of the tooth shape. With conventional standard brackets, there was also the formation of edge cracks, but there were not as many areas per bracket as in the APC flash-free brackets and therefore a lower level of bacteria.

In terms of further clinical potential the studies by Julien et al. (2013), H. E. Kim et al. (2015), and J. Kim et al. (2016) showed that there is great potential for the minimization of white spot lesions during orthodontic therapy with a multibracket appliance. Julien et al. found that 23.4% of patients developed white spot lesions during multibracket therapy [5]. The study by H. E. Kim et al. (2015) showed how the white spot lesions are visualized by spectrophotometer and there are probably more than the intraoral clinically visible lesions and enamel changes [20]. Enamel changes can occur during and after orthodontic therapy as well as also due to enamel avulsion as microleakage after debonding, which was studied by J. Kim et al. in APC flash-free brackets [30]. For these reasons, the bacterial formation in the bracket environment is clinically relevant to the demineralization processes of enamel and the development of white spot lesions. However, when the attachment of conventionally bonded standard brackets is thoroughly processed, the use of very little excess adhesive is achievable. Nevertheless, this standard technique is more time-consuming, and the effective removal of excess is not possible at any time at all points of the bracket during the direct intraoral attachment of multibracket appliances [16, 24, 31, 32].

## Conclusion

The excess adhesive after bracket bonding has an influence on the subsequent bacterial adhesion. Bacterial storage was significantly higher in the adhesive range of the standard brackets. A smooth adhesive closure with a small excess of adhesive has a positive effect but can lead to a risk of margin edge gaps with bacterial colonization, as shown with the APC flash-free brackets.

## Data Availability

The datasets of this study are available upon reasonable request from the corresponding author.
